# Tilt perception is different in the pitch and roll planes in human

**DOI:** 10.14814/phy2.15374

**Published:** 2023-02-13

**Authors:** Dimitri Keriven Serpollet, David Hartnagel, Yannick James, Stéphane Buffat, Nicolas Vayatis, Ioannis Bargiotas, Pierre‐Paul Vidal

**Affiliations:** ^1^ Training & Simulation, Thales AVS France SAS Osny France; ^2^ Centre Borelli, Université de Paris, ENS Paris‐Saclay, CNRS, SSA Paris France; ^3^ Département Neurosciences et Sciences Cognitives Institut de Recherche Biomédicale des Armées Brétigny‐sur‐Orge France; ^4^ Laboratoire d'Accidentologie de Biomécanique et du comportement des conducteurs GIE Renault‐PSA Groupes Nanterre France

**Keywords:** full flight simulator, motion perception, otoliths, perceptual threshold, pilot, semicircular canal, vestibular perception

## Abstract

Neurophysiological tests probing the vestibulo‐ocular, colic and spinal pathways are the gold standard to evaluate the vestibular system in clinics. In contrast, vestibular perception is rarely tested despite its potential usefulness in professional training and for the longitudinal follow‐up of professionals dealing with complex man–machine interfaces, such as aircraft pilots. This is explored here using a helicopter flight simulator to probe the vestibular perception of pilots. The vestibular perception of nine professional helicopter pilots was tested using a full flight helicopter simulator. The cabin was tilted six times in roll and six times in pitch (−15°, −10°, −5°, 5°, 10° and 15°) while the pilots had no visual cue. The velocities of the outbound displacement of the cabin were kept below the threshold of the semicircular canal perception. After the completion of each movement, the pilots were asked to put the cabin back in the horizontal plane (still without visual cues). The order of the 12 trials was randomized with two additional control trials where the cabin stayed in the horizontal plane but rotated in yaw (−10° and +10°). Pilots were significantly more precise in roll (average error in roll: 1.15 ± 0.67°) than in pitch (average error in pitch: 2.89 ± 1.06°) (Wilcoxon signed‐rank test: *p* < 0.01). However, we did not find a significant difference either between left and right roll tilts (*p* = 0.51) or between forward and backward pitch tilts (*p* = 0.59). Furthermore, we found that the accuracies were significantly biased with respect to the initial tilt. The greater the initial tilt was, the less precise the pilots were, although maintaining the direction of the tilt, meaning that the error can be expressed as a vestibular error gain in the ability to perceive the modification in the orientation. This significant result was found in both roll (Friedman test: *p* < 0.01) and pitch (*p* < 0.001). However, the pitch trend error was more prominent (gain = 0.77 vs gain = 0.93) than roll. This study is a first step in the determination of the perceptive‐motor profile of pilots, which could be of major use for their training and their longitudinal follow‐up. A similar protocol may also be useful in clinics to monitor the aging process of the otolith system with a simplified testing device.

## Graphical abstract

Vestibular perception is rarely tested despite its potential usefulness in professional training and for the longitudinal follow‐up of professionals dealing with complex man‐machine interfaces, such as aircraft pilots. In this study, we tested the motion perception in the dark of professional helicopter pilots in a full‐flight simulator. We showed that they are less precise in the sagittal plane than in the frontal plane.

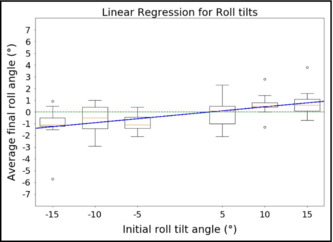

**News and Noteworthy**

To the best of our knowledge, there have been few empirical studies comparing the frontal and sagittal planes of space. In this study, we demonstrate the prominent role of utricles in verticality and/or horizontal perception and how it has a different behavior in frontal (operating in a pull‐push fashion) and sagittal (acting synergistically) planes.

## 1 INTRODUCTION

The perception of body orientation is based on the integration of gravity‐based visual, vestibular, and somesthetic information. However, the respective contribution of these sensory systems (i.e. the weight attributed by the central nervous system during the integration process) is still debated. As the topic of our study is the role of the vestibular system in movement perception, we will only debate the respective contribution of the vestibular and somesthetic information.

On the one hand, electrophysiological recordings of the discharge of primary otolith neurons in response to head tilts (Fernandez & Goldberg, 1976; Jamali et al., 2009; Sadeghi et al., 2007) demonstrate that the otoliths signal both the static orientation of the head to gravity, but also changes in orientation that accompany angular head displacements (Benson, 1990). Also, following unilateral vestibular global and selective lesion, in rodents, their posture remains permanently tilted despite the compensation process, showing that asymmetric otolithic information remains asymmetric despite the persistence of symmetric somesthetic information. In humans, vestibular thresholds have been well studied (see Kobel et al., 2021 for a recent review), especially the roll tilt thresholds (Hartmann et al., 2014; King et al., 2019; Lim et al., 2017; Valko et al., 2012). Rosenberg et al. (2018) demonstrated a relationship between an individual's roll tilt vestibular perceptual threshold and their performance in a manual control task. As pointed by the authors, this suggested that sensory precision was a critical determining factor in manual control performance. The correlation between manual control performance and threshold suggested that vestibular precision determined performance. Vestibular migraine patients were shown to be abnormally sensitive to roll tilt, which co‐modulates semicircular canal and otolith organ activity (King et al., 2019). Israel and Berthoz (1989) submitted human subjects to horizontal linear displacements along the interaural (*Y*)‐axis in darkness, seated in a cart moving along a linear track. They found that an approximate estimation of head displacement can be derived from the linear acceleration measured by the otoliths. Indeed, bilabyrinthectomized patients could not perform gaze stabilization or approximate head displacement. This shows that the observed performance was of vestibular origin. Also Walsh (1961) reported translation thresholds that were about 10 times greater than normal in deaf students without evidence of an angular vestibulo‐ocular reflex (VOR). Furthermore, Valko et al. (2012) to assess the contributions of the vestibular system to whole‐body motion discrimination in the dark, measured direction recognition thresholds as a function of frequency for yaw rotation, superior–inferior translation (“z‐translation”), interaural translation (“y translation”), and roll tilt for normal subjects and patients following total bilateral vestibular ablation. The patients had significantly higher average threshold measurements than normal for every rotation tested, including roll tilt (Suri & Clark, 2020) investigated the difference between pitch tilt thresholds versus roll tilt thresholds using vestibular psychophysics. Using a general linear model, they found that pitch tilt thresholds were significantly higher than roll tilt thresholds across a range of frequencies (from 0.15 to 1 Hz). This result is supported by other works (Israël & Giannopulu, 2012; Karmali et al., 2021). Altogether, it appears that one could probe otolith contribution to body orientation using perceptual threshold below the threshold of activation of the semicircular canal, despite the inherently multimodal nature of movement detection.

On the other hand, experiments conducted in microgravity emphasized the importance of some somesthetic cues for the perception of body orientation (Lackner & DiZio, 1993, 2000). Gianna et al. (1996) reported just a slight increase in the translation thresholds of vestibular defective subjects. During immersion and in a buoyant state, the somesthetic system has limited access to gravity‐based information, whereas gravity still acts on a component of the vestibular system. Subjects' postural responses to the vertical underwater were strikingly inaccurate, showing a forward tilt ranging from 7.3° Massion et al. (1995) to 13.2° Ross et al. (1970). This suggests that gravity‐based somesthetic cues may play also a more important role in perceiving a static body orientation. Bringoux et al. (2003) showed that the threshold for the perception of a body tilt was higher when subjects were completely immobilized in a body cast than when partially restrained. Hence, these authors conclude that for very slow velocities, signals issued from the vestibular system were not naturally efficient in quasi‐static conditions for accurately perceiving the body orientation with respect to the gravity field. On the other hand, somesthetic information such as tactile, proprioceptive (Higashiyama & Koga, 1998), and interoceptive (Mittelstaedt, 1992) cues played a prominent role in postural orientation.

The raison d'être for the perception of body orientation based on the integration of gravity‐based visual, vestibular, and somesthetic information is that precise and accurate motion control is mandatory for survival in day‐to‐day life and even more so in aircraft pilots. However, sensorimotor responses and perception are intrinsically imprecise because of noise in neural systems (Faisal et al., 2008). Indeed, motor variability in the vestibulo‐ocular reflex evoked during yaw rotation in rhesus monkeys (Haburcakova et al., 2012) and humans (Nouri & Karmali, 2018; Seemungal, 2014) are similar to human perceptual yaw rotation thresholds suggesting a common, sensory source of the noise.

In that context, movement perception based on vestibular and somesthetic cues is valuable not only for the diagnosis of vestibular and somesthetic pathologies but also for the longitudinal follow‐up of complex man–machine interface operators such as plane and helicopter pilots, where it becomes a matter of survival. Indeed, aircraft pilots are subject to many situations where they have to orient themselves without any visual cues. For instance, during high altitude night flights, plane pilots often use their flight instruments over visual (from the outside view) or sensory cues to pilot their aircraft. Another example specific to helicopters is a phenomenon called Rotary‐Wing Brownout (RWB) and Whiteout. As described by Priot (2012), a brownout is the condition developed by recirculating rotor downwash as helicopter lands or takes off in an arid or a snowy (whiteout) environment. The dust, dirt, or snow that is developed by the downwash renders out‐the‐cockpit visibility severely degraded or non‐existent. This phenomenon will reduce the number of visual cues available for the helicopter crew. This loss of visual cues can also happen during instrument meteorological conditions (IMC) when there are poor weather conditions. In those situations and aviation in general, the vestibular system could become a crucial player in the pilot's situational awareness, notably to characterize self‐motion and to differentiate passive from active head motion (Cullen, 2014) and a key element in the decision‐making process. On the one hand, in the limited case of rotary‐wing take‐off or landing, vestibular cues could be somewhat useful. On the other hand, for coordinated flight in a fixed‐wing or rotary‐wing aircraft, relying on vestibular cues could be a deadly mistake. Note that these points will not preclude the utility of testing regularly motion perception of pilots. An abnormal aging process and/or a more or less well‐compensated pathology of the vestibular system could still introduce a supplementary bias in pilots' perception. Finally, vestibular‐induced spatial disorientation and illusions such as the leans, the graveyard spin, the graveyard spiral, the Coriolis inversion, head up and head down illusions are a significant cause of crashes. In accidents involving disorientation, 85% are a consequence of unrecognized disorientation during complex flight scenarios (Stott, 2013).

As underlined above, except in rare cases of specific pathologies, one can hope at best to disentangle the confounding influence of the various source of sensory information when assessing movement perception. This plead in favor of the development of psychometric tests in realistic situations, particularly for the monitoring of aircraft pilots, as self‐perception is context‐dependent but vestibular thresholds do not probe extra vestibular sensory information.

Nevertheless, to the best of our knowledge, only a few studies focused on that topic using flight simulators (Heerspink et al., 2005; Hosman & Van der Vaart, 1978; Zaichik et al., 1999) or using both simulator and real aircraft (Tribukait, Bergsten, et al., 2016; Tribukait, Ström, et al., 2016). It was shown in those studies that the yaw threshold is the greatest among yaw, pitch, and roll thresholds. Therefore, the question arises whether the wealth of psychophysical data accumulated in naïve or pathological persons using rigorous psychophysical tests in laboratory environment transfer to operators of complex Human‐machine interface, such as aircraft pilots. To the extent we specifically used the most realistic flight simulator, we expect that our findings will generalize to tilt perception in the context of real air flight operations. This would justify the use of regular testing of the vestibular function of pilots during their routine on simulators. It also opens the question of the context‐dependency of human motion detection in realistic situations. To tackle these questions, we investigated ego‐motion perception in a professional helicopter using a flight simulator. We first focused on the role of the otoliths and somesthetic contribution on the outbound displacement by taking advantage of the fact that for small tilts (approximately 10°) stimuli below 0.1 Hz, among the vestibular sensors, the otolith organs would play a prominent role in motion detection. In contrast, higher frequency stimuli are detected by semicircular canals (Nashner, 1971; Ormsby & Young, 1977). In addition, the velocities of the outbound displacement of the cabin were performed at a range of velocities, which are below the average threshold of the semicircular canals (1.5 °/s, Lee et al., 2020).

## 2 MATERIALS AND METHODS

### 2.1 Subjects

Nine males operational professional helicopter pilots aged between 31 and 48 (39 ± 5.7) years participated in this study. They had no known history of balance impairment or dizziness. These pilots had between 300 and 5000 h of helicopter flights to their record (all aircraft combined). The Institutional Review Board Paris Descartes (CERES N°2017‐35 dated 23/5/2017) approved the experimental protocols following the 1964 Helsinki Declaration.

### 2.2 Experimental setup

#### 2.2.1 Simulator

**FIGURE 1:**
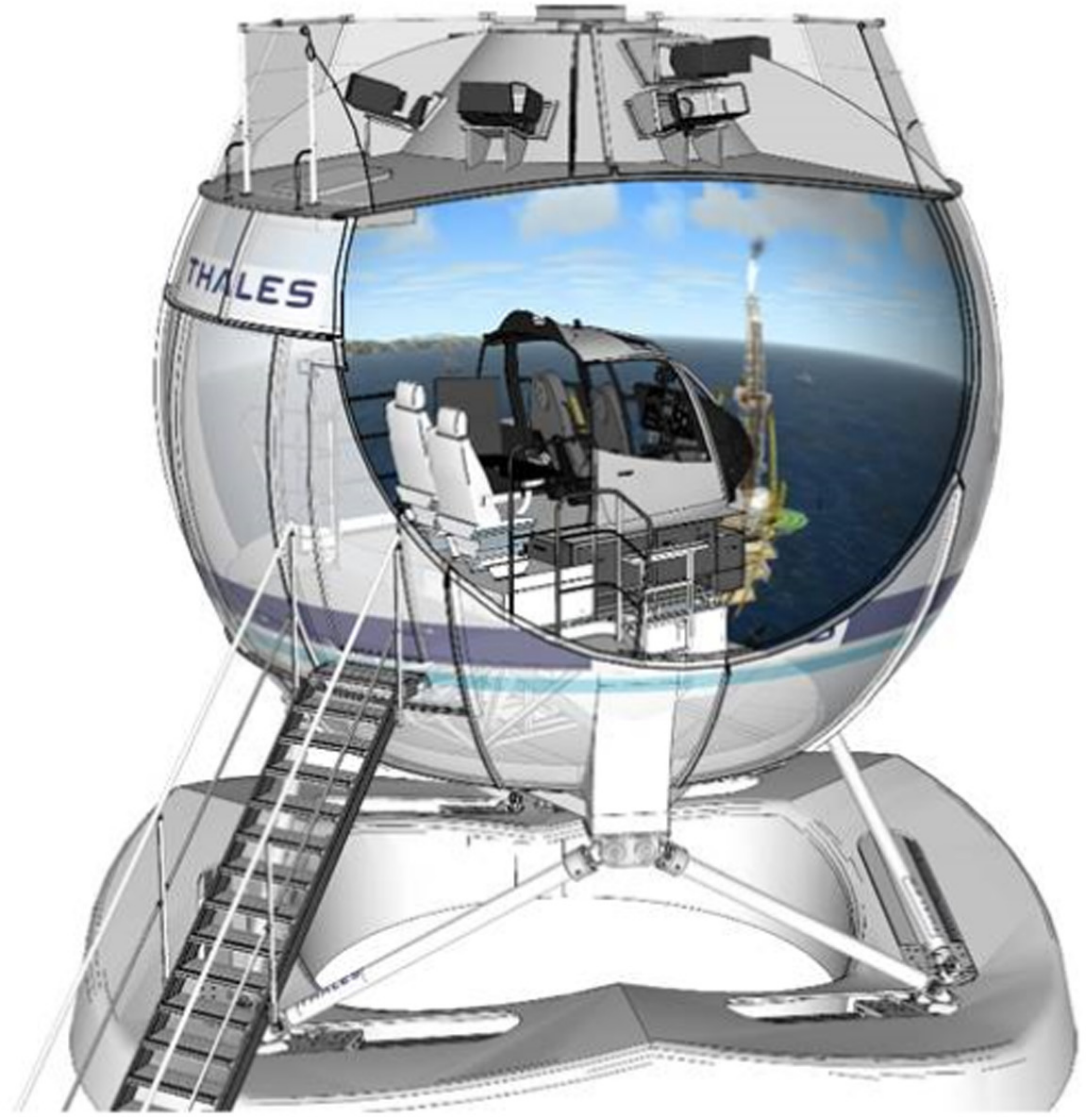
The EC135 cabin is in the display dome and the dome is over the six‐degrees‐of‐freedom motion platform. The pilot is seated in the cabin.

All experiments were performed on a helicopter simulator illustrated in Figure 1 that included a motor‐driven support surface and visual surround (Figure 1). Position servo‐controlled motors produced anterior/posterior (AP) tilts of the support surface and visual surround with the rotation axes centered on the head of the subject and collinear with the horizontal plane without any tilt. Level‐D is the highest level of certification and the only level qualifying for ZFT (“Zero Flight Time”) training. The simulator is equipped with an EC135 (Eurocopter) helicopter cabin. A joystick (Logitech Extreme 3D pro [X3D]) is used by the pilot to move the cabin rather than the helicopter commands. A specific software was developed to drive the motion platform with the joystick. The simulator was programmed so that there was a linear relationship between the motion of the joystick and rotation speed. The two axes of the joystick were used to have a natural gesture to move the cabin: forward/backward to move in pitch and left/right to move in roll. To move the simlulator in “yaw”, the pilots could move the joystick in the horizontal plane. Before the experiment, all pilots went through a realistic flight simulation to get used to this specific simulator.

#### 2.2.2 Random stimuli and protocol

**FIGURE 2:**
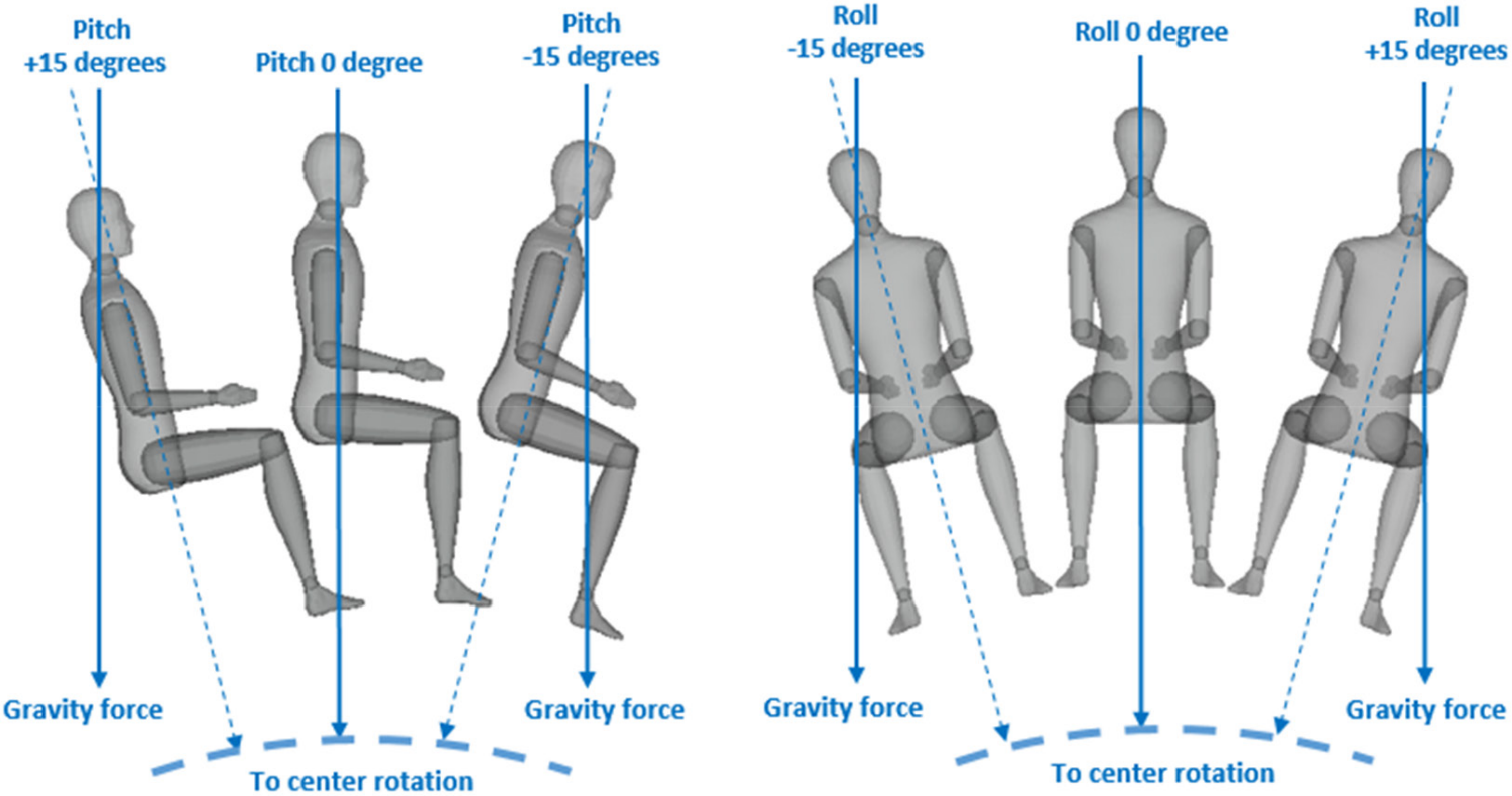
Examples for the ±15° pitch and roll tilts. All tilts were centered 2.6 m below the pilots' heads.

During the whole experiment, the pilots were sitting in the level‐D full flight simulator in complete darkness. All visual cues were shut down and the lights were switched off. Then the simulator's cabin tilted 14 times: 6 times in frontal plane (roll tilt), 6 times in sagittal plane (pitch tilt), and two times in the horizontal plane (yaw rotation). The tilts were centered 2.6 m below the pilots' heads (see Figure 2). The yaw rotations were −10° and 10°. Both roll and pitch tilts were −15°, −10°, −5°, 5°, 10°, and 15°, with a maximum speed of 0.85 °/s and a maximum acceleration of 0.2°/s^2^. The sequence of the 14 tilts was randomized. The cabin's velocities displacement were below the threshold of the semicircular canals (1.5°/s, Lee et al., 2020) and lasted between 10 and 20 s. After the completion of each cabin tilt, the pilots were asked to put the cabin back into the horizontal plane (i.e. in the neutral position) without any visual cue or time constraint. The return operation can be done with a maximum speed of 1.7 °/s. To ensure the pilots would not damage the equipment, the cabin could only move in the plane of the previous passive movement. For instance, if the cabin tilted at 10° in the sagittal plane initially, then the pilot could only move the cabin within the sagittal plane too. At the time the pilot thought he was indeed back in the neutral position, he had to press a button. Then the cabin was repositioned in its neutral position and the next tilt occurred with a latency of 45 s. If the pilot did not attempt to put the cabin back to the neutral position within 45 s, the cabin would do so automatically. The experiment lasted about 20 min for each participant. Before testing, all participants gave their informed consent.

For the initial inclination of the simulator, we desired to minimize the somesthetic and semicircular canals information, as our goal was to measure the contribution of the otolith to motion perception. Due to severe time constraints of the professional pilots, we were unable to measure the canal threshold for each participant. Therefore, we decided to limit our peak velocity to 0.85 °/s for all participants, based on the semicircular canal threshold of the study of Lee et al. (2020); in this study, the authors found that the canal threshold is 1.5 °/s on average (for 1 Hz cycles) but the range goes actually from 0.69 to 2.99 °/s with only one of the 15 participants having a threshold below 0.8 °/s. Also, according to Grabherr et al. (2008) the semicircular canal threshold achieves a plateau at 1 Hz around 0.71 °/s (see Figure 2 of the paper), which is in accordance with Lee et al. (2020). Moreover, Grabherr et al. (2008) found that the semicircular canal threshold increases from 0.85 to 3 °/s when the frequency decreases from 1 to 0.05 Hz. Our test's frequency is around 0.02 Hz. Altogether then, we felt confident that by keeping our peak velocity below 0.85 °/s we did not stimulate the semicircular canals.

On the other hand, we did not impose a velocity limitation for the return trajectories of the simulator. The reason was that we wished the pilots to be as precise as possible when they repositioned the simulator to its initial position, by using all sensory information available, including the one from the semicircular canals.

Concerning the other potential cues: (1) all the screens and flight instruments were shut down and as the simulator cabin is a closed environment, the darkness was maximal; (2) the only auditory cue available was the sound of the motors moving the simulator, which was exactly the same in every direction (pitch, roll or yaw) and was not modulated by the position of the cabin.

## 3 DATA ANALYSIS

### 3.1 Measurements

The pilots did not have a time limitation to perform their task. Hence, the only factors that were taken into account for further analysis were the initial passive tilt angle imposed on the cabin and the accuracy of the pilot in recovering its neutral position. We defined the accuracy as the tilt angle of the simulator once the pilots considered they have achieved to reposition the cabin in its initial position.

### 3.2 Statistics

For each pilot, we calculated the average accuracy of the repositioning of the cabin in the neutral position following the yaw, pitch, and roll tilts. Wilcoxon signed‐rank test was used to perform a paired difference test between overall roll and pitch tilts. It was used over a Student *t*‐test, as the distribution of the roll samples could not be assumed to be normally distributed. The Shapiro–Wilk test (to test normality) returned a normal distribution for the pitch samples (*p* = 0.335) while a non‐normal distribution for the roll samples (*p* = 0.004). Friedman test was used to evaluate the trials' accuracy with respect to the initial tilt angle and to find potential trends or relations between the accuracy and the initial tilt angles.

### 3.3 Analysis

All data analyses were performed using the latest version of Python (3.10.0) through Jupyter Notebook.

## 4 RESULTS

The average accuracy (across all nine pilots and all trials) for the yaw movements amounted to 10.6° ± 3.6° and 8.7° ± 2.7° for the right and left yaw movements of the cabin. The cabin was moved at random either 10° to the right or 10° to the left, and none of the pilots were able to retrieve the straight‐ahead direction following the cabin displacement in yaw. It was found that only five of the 18 yaw trials resulted in the pilots attempting but ultimately failing to move the simulator back to its neutral position. In the remaining 13 trials, either the pilots did not notice the cabin movement or renounced to correct its orientation once it was completed due to insufficient information on their passive displacement.

**FIGURE 3:**
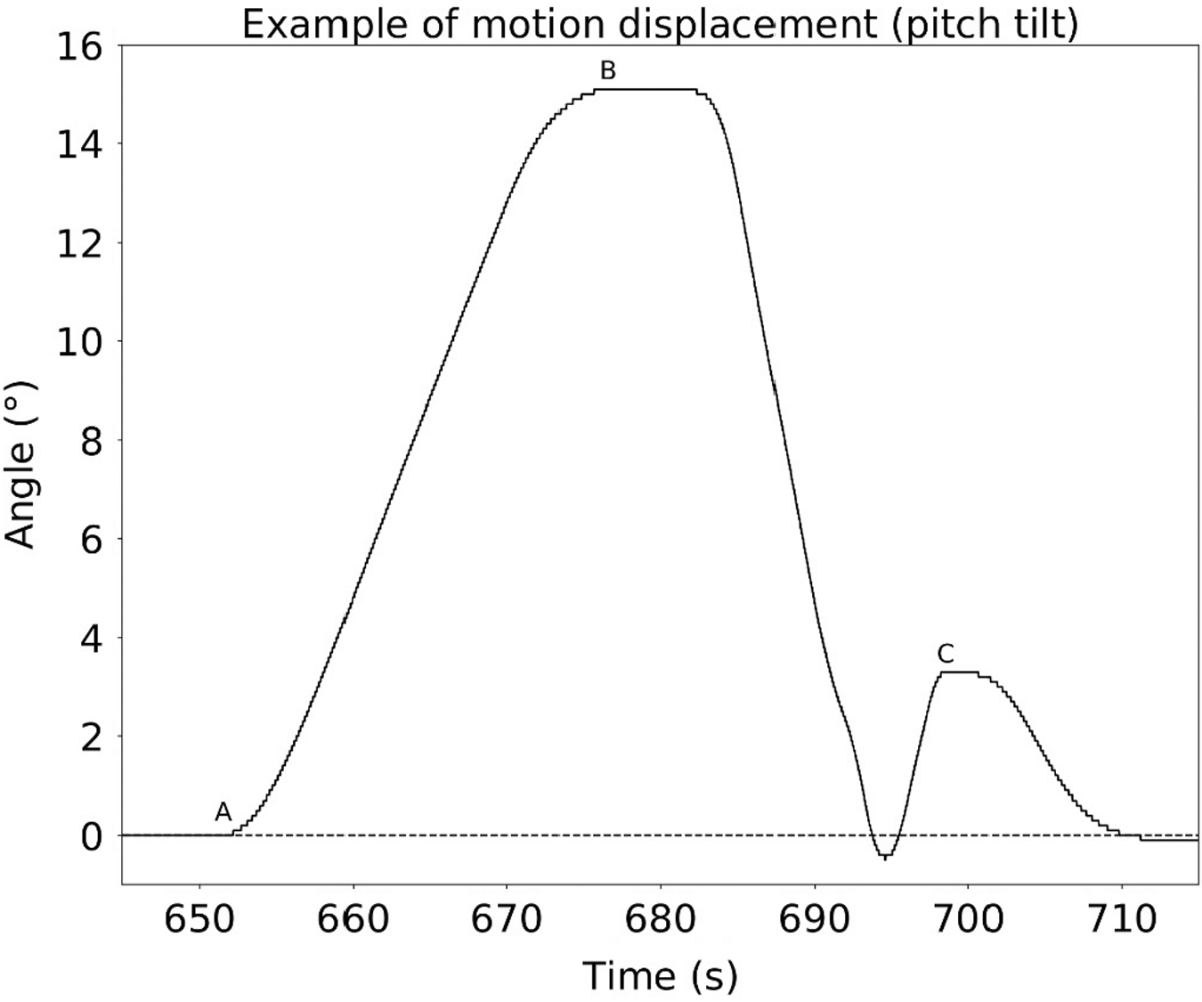
Example of motion displacement of the simulator cabin in the sagittal plane. There are three key moments: (a) Start of the outbound motion displacement toward 15°; (b) End of the outbound motion displacement, the pilot is now able to move the simulator; (c) The pilot stops moving the simulator, presses the button, and a few seconds later, the simulator goes back automatically to the neutral position on its own.

Figure 3 below displays a typical angle displacement of the simulator cabin in the sagittal plane.

The average absolute accuracy (across all nine pilots and all trials) for the roll tilts was 1.15° against 2.89° for the pitch tilts. Wilcoxon sign‐ranked test showed that pilots were significantly more precise in roll tilts (*p* < 0.01). However, within planes, no difference was found between (i) left and right roll tilts; (ii) forward and backward pitch tilts. Both *p*‐values are greater than 0.05 (respectively, 0.48 and 0.15).

Table 1 below summarizes those results: *p*‐values and average values (mean and SDs).

**TABLE 1:** Mean and standard deviation of accuracy and p‐value of Wilcoxon signed‐rank test at given conditions

	Mean	Standard deviation	Wilcoxon *p*‐value	Statistic value
Roll (average)	1.15°	0.67°	<0.01	0
Pitch (average)	2.89°	1.06°		
Left Roll	1.35°	1.20°	0.4838	13
Right Roll	0.94°	0.54°		
Forward Pitch	2.38°	0.82°	0.1548	10.5
Backward Pitch	3.42°	1.92°		

**FIGURE 4:**
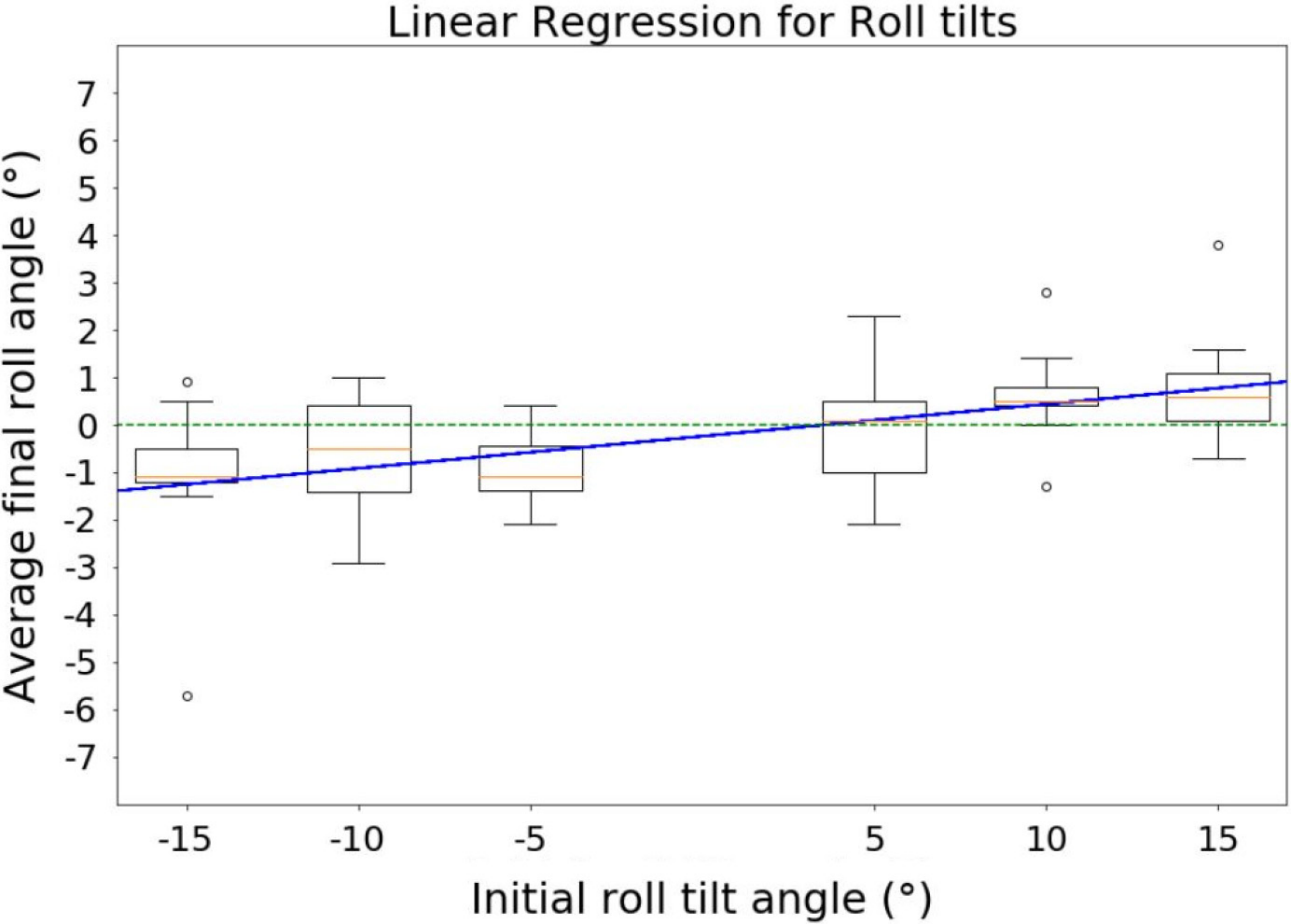
Trend of accuracy average for roll tilts. Negative tilts are left roll tilts; positive tilts are right roll tilts. This figure shows the trend of the accuracy average with respect to the initial tilt angle (averaged across every pilot) for roll tilts only. Even though the slope of the linear regression is small (0.068), we can see a clear tendency to bias with respect to the initial tilt: The greater the initial tilt angle, the less precise the pilots were, although maintaining the direction of the initial tilt. Furthermore, a Friedman test showed this bias is statistically significant (*p* < 0.01).

**FIGURE 5:**
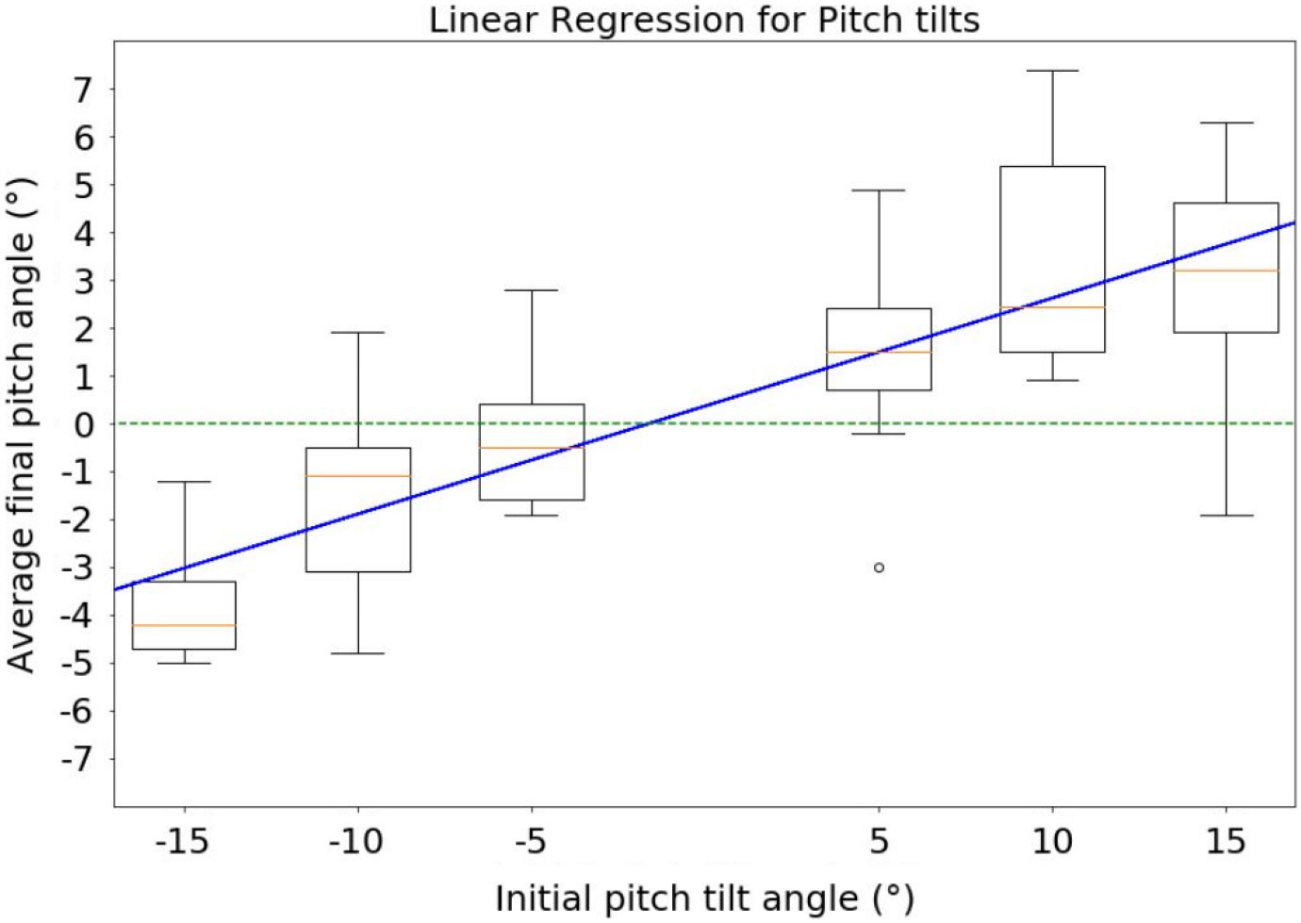
Trend of accuracy for pitch tilts. Negative tilts are backward pitch tilts; positive tilts are forward pitch tilts.This figure shows the same thing as Figure 4 but for pitch tilts. The result is the same but more prominent, here the slope of the linear regression is 0.22. A Friedman test also showed a statistically significant bias toward the initial tilt angle (*p* < 0.0001).

Furthermore, as illustrated in Figures 4 and 5, the analysis of the accuracy with respect to the initial tilt angles (−15°, −10°, −5°, 5°, 10° or 15°) showed that the greater the initial tilt was, the less precise the pilots were, although maintaining the direction of the tilt. A Friedman test was performed and showed that differences within the same plane were statistically significant (*p* < 0.01 for roll tilts and *p* < 0.0001 for pitch tilts).

Finally, linear regressions were performed to address whether there was a linear relationship between the initial tilt angle and the accuracy of pilots. Considering the size of the population (9 pilots), the coefficient of determination for both regressions was acceptable (0.92 for roll tilts, 0.94 for pitch tilts). Furthermore, the slopes showed that the pitch trend (slope = 0.23, gain = 0.77) was more prominent than the roll trend (slope = 0.068, gain = 0.93). Figures 4 and 5 show the results of the linear regression, respectively, for roll and pitch tilts. Table 2 summarizes the results for this analysis (slopes and coefficient of determination). Table 3 summarizes the statistics of the Friedman test.

**TABLE 2:** Linear regression results

	Roll tilts	Pitch tilts
Coefficient of determination	0.92	0.94
Slope	0.068	0.22
*p*‐value	0.002	0.001

**TABLE 3:** Friedman test results

	Roll tilts	Pitch tilts
*p*‐value	<0.01	<0.0001
Test statistic	19.05	33.82

## 5 DISCUSSION

In this study, we measured the accuracy of nine professional helicopter pilots who were asked to put a level‐D Full‐Flight Simulator back in its neutral position after having been tilted in a pitch or roll direction at an unknown given angle without any visual cues. It appears that the pilots were significantly less precise in pitch tilts (sagittal plane) than in roll tilts (frontal plane). Furthermore, the pilots were biased toward the initial tilt angle. We found that the greater the initial tilt angle was, the less precise the pilots were. This result was found for both pitch and roll tilts, even though the effect was more prominent in pitch tilts than in roll tilts.

### 5.1 Respective contributions of otoliths and proprioceptive information in movement detection in our protocol

Auditory, vestibular, proprioceptive, and visual information could have played a role in the detection of the movement of the cabin (Bronstein, 1999; Kavounoudias et al., 1999; Roll et al., 2002). However, some of these cues were excluded (visual information), minimized (tactile feedback by covering their skins), or controlled (auditory). It remains that proprioceptive information could not be completely ruled out. Indeed, professional pilots are notorious to use proprioceptive cues to disambiguate their motion perception in case of sensory conflicts, including somatic trunk graviception (Rupert, 2000). On the other hand, we did not observe differences in the pilots' performance between backward and forward pitch tilts despite the large difference in the proprioceptive information generated in both cases (there was no headrest). In addition, pilots could not orient themselves during the yaw stimuli when vestibular information was absent. Hence, proprioceptive input and auditory information probably played a minor role in our task.

Concerning the vestibular information, both semicircular canals and otoliths were a putative source of information. Nevertheless, a major contribution of the semicircular canals' information on the outbound displacement can probably be ruled out for three reasons. First, the cabin movements stimuli were delivered below 1.5 °/s, which is below the average threshold of the semicircular canal activation (Lee et al., 2020). Second, the power spectrum of the cabin movements did not encompass any frequencies above 0.02 Hz; below this frequency, the otoliths play a prominent role in movement perception (Nashner, 1971; Ormsby & Young, 1977). Third, the cabin movements in yaw, which could only generate semicircular canal information, were not perceived. Furthermore, according to (Benson et al., 1986) the linear acceleration threshold in the horizontal plane is 0.05 m/s^2^ for the otolith organs. In our case, the linear acceleration is 0.01 m/s^2^; thus the otolith organs did not perceive the linear component of the cabin movement.

Altogether, otolith information appears to be important for self‐motion perception. Along that line of thought, postural control in patients with unilateral vestibular lesions was more impaired in the roll than in the pitch plane (Mbongo et al., 2004, see also Pavlovic, 2019). Furthermore, guinea pigs with a unilateral lesion of the utricle displayed a lateral whole‐body tilt in the frontal plane and a forward tilt in the sagittal plane following a bilateral utricular lesion (De Waele et al., 1989).

### 5.2 Why a bias in the perception of ego‐motion by the utricular system?

What could be the cause of the perceptual asymmetry we observed in the roll and pitch tilts? Several non‐mutual explanations can be offered. First, the utricle plays a prominent role in the perception of verticality, and it could mean that self‐verticality is transduced in the brain by a null difference between the left and right utricles. Considering the movements of the simulator were constrained in the sagittal or the frontal plane, it shows that the null difference was more accurate when the simulator was moving in the frontal plane, i.e., when the two utricles were functioning in push‐pull rather than in the sagittal plane when they acted synergistically.

Second, Suri and Clark (2020) proposed a hypothesis involving the geometry of the otolith organs. The utricular maculae are about level in the frontal plane, while pitched up about 30° in the sagittal plane. Thus, tilts produce a larger change in the utricular shear stimulation for roll tilt compared to pitch tilt. It remains that the anatomical orientation of the otolith organs should also induce a difference between forward and backward pitch tilt, which was not found.

Third, the human body has a higher number of degrees of freedom available in the sagittal plane than in the frontal plane at the spine, knees, hips, and neck level. As a result, abundant sources of proprioceptive information are available to document ego‐motion perception in the sagittal plane. This would be less so in the frontal plane, and therefore the role of the utricular information would be more prominent.

Heerspink et al. (2005) using aircraft coordinated turns during real flights and centrifuge, investigated selectively the role of the semicircular canals' information in pilots' self‐motion perception. They found a pronounced underestimation of pitch and roll angular displacements. The interindividual variability was also considerable. That is, in aircraft pilots, both semicircular canals and otoliths provided information that is biased and underestimated. It likely contributes to the disorienting movement patterns that are commonly reported during flight.

One particularly relevant area of prior research concerns the so‐called subjective postural vertical (SPV). Notably, Bergmann et al. (2020) found that SPV assessment while sitting induced a larger SPV range compared to the assessment while standing, indicating larger insecurity in verticality estimation while sitting. These authors attribute that fact to a higher availability or reliability of somatosensory inputs while standing. Interestingly, it could suggest that pilots rely more on the vestibular cues due to their sitting position, which pleads in favor of regular vestibular testing. Moreover, in good accordance with our results, Bergmann et al. (2020) found that the range of SPV determination is larger in pitch than in roll. The difference, in our results, in the accuracy in the roll and pitch planes, is likely due to different methods (we took the absolute error while Bergmann et al., 2020 did not) which makes the comparison difficult.

Finally, we did not find any clear relationship between our results and the flying experience or the age of the participants.

### 5.3 Should the vestibular function of pilots be tested on a regular basis?

Altogether, one can test the motion perception of professional aircraft pilots using real‐flight, aircraft simulators, and centrifuges. In particular, a test of the semi‐circular canals and the otolith test we described here could be implemented in the mandatory simulator sessions imposed on pilots every 6 months. In support of that proposal, several arguments can be offered: first, the large inter‐individual differences we and others observed in the tested pilots: for instance, one pilot was the worst performer than the others, although his age and flight experience were in the average of our sample. Second, the career of a pilot spans over several decades, and the sensory systems age (Boisgontier et al., 2012; Brosel et al., 2016; Sloane et al., 1989). Third, during training or later in professional life, perceptual learning is feasible in all sensory modalities (Ertl et al., 2020). Fourth, there is potentially a relationship between vestibular functions and manual control tasks in piloting. Indeed, Rosenberg et al. (2018) studied a “vestibular” manual control task in which subjects attempted to keep themselves upright with a joystick to null out pseudorandom, roll‐tilt motion disturbances of their chair in the dark. They found a significant correlation between subjects' vestibular perceptual thresholds and performance in a manual control task, consistent with sensory imprecision negatively affecting functional precision. Fifth, an abnormal aging process and/or a more or less well‐compensated pathology of the vestibular system could bias pilots' perception as clinically observed in vestibular patients (Zalewski, 2015).

An increase of 0.2 Hz roll tilt threshold predicts more than a six‐fold increase in fall risk (Agrawal et al., 2009; Bermúdez Rey et al., 2016; Beylergil et al., 2019; Karmali et al., 2017). That is, our study also suggests that probing movement perception in roll and pitch using a simpler device than the flight simulator would be also useful to identify age‐related vestibular function decline and fall risk, as also underlined in the review of (Kobel et al., 2021).

### 5.4 Study limitations

Our study has some limitations that narrow the scope of this paper. First and foremost, there is no control group: the only subjects involved are professional helicopter pilots, a population that is likely to have a highly trained vestibular system. Thus, it is questionable whether our results apply as such to the general population. Unfortunately, we are not able to perform any new measures due to the high cost and the affordability of the equipment used to perform this experiment. If our proposal that vestibular testing in the routine of pilots is accepted we will indeed have to perform another set of control experiments.

Also, during the tilts of the cabin and the return trajectories, we cannot exclude the participation of the proprioceptive information. Moreover, due to time constraints, we were not able to explicitly assay the perception through a rigorous psychophysics test to measure the precise semicircular canal thresholds for each pilot for instance.

We also had a constraint on the simulator displacements: to enhance the safety of the experiment, the pilots could only move the cabin in the plane of space along the direction it was passively tilted. This could have biased our dataset. Indeed, only the simulator was constrained in the given plane, the pilots were able to move the joystick in any direction.

We also chose different velocities for the outbound and the return of the simulator. For the outbound, the velocity was chosen to test the vestibular otolith system; on the return, we felt that it was important that the pilots were free to choose their own velocity to not bias the accuracy of their estimation of their passive displacement.

Finally, the possibility that the pilot who performed worse than the others is an outlier remains. For instance, a lack of selective attention could be at play.

## AUTHOR CONTRIBUTIONS

Dimitri Keriven Serpollet: analyzed the data and wrote the manuscript. David Hartnagel, Yannick James and Stéphane Buffat: designed and performed the experiments. Nicolas Vayatis: secured the funding. Ioannis Bargiotas: supervised the data analysis. Pierre‐Paul Vidal: secured the funding, helped for the experimental design, and supervised the manuscript writing.

## CONFLICT OF INTEREST

The authors report that they studied the pilots of a flight simulator rendered available by Thales, which is also the employer of two of the co‐authors of the study: D. Keriven Serpollet and Y. James.

## ETHICAL APPROVAL

All procedures and protocols used in this experiment were approved by the Institutional Review Board Paris Descartes (CERES n°2017‐35 dated 23/5/2017) in accordance with the 1964 Helsinki Declaration.
